# Signs of the Times

**Published:** 1886-11

**Authors:** 


### SIGNS OF THE TIMES.

In the mid'st of theological squabbles which carry one back through darkness, to their middle-age likenesses, it is encouraging to patient and suffering humanity, to find in the columns of so conservative an organ as the "*Church Union*," the following communicated article upon the subject of

### "CREEDS."

"Creeds are made to cut off Christians. Even in *confessions*, they are confessions to the popular party, that the adherents are not "like others who are thus sacrificed for the favor of the great." So Servetus went to the flames.

Creeds cramp thought. They forbid the growth of any mind beyond their "Dead Line;" on pain of spiritual death.

Creeds limit progress and growth. "Their very lesson is to lead the young disciple back to the grave of the old school of thought.

Creeds imprison the mind. "Their intent is to prevent freedom. The defence is "If we do not make the creed authorative, coming generations will forsake their dogmas." That means "We will place the shackels on the thought of coming generations and blind the sight of others."

Creeds are unjust. It is admitted that we have more light and Bible knowledge than the dark ages, consequently we can better make creeds for ourselves than they for us.

*Creeds are untrue.* Their statements are so evidently untrue, and so contradict each other that their defenders fly to mystery for defence.

*Creeds* have caused more harm to the church than all the outward persecution it has ever endured. The millions slain in their defence is not a moiety of the evil they have done.

Creeds are a standing disgrace to the church as there is no well known or leading creed of human origin from the Nicene to the Athanasian that is believable or biblical, "except in parts."

A western religious conference of the fossilated school of thought has recently had under serious discussion the question, as to whether the heathen, in their benighted state, who never heard and never could hear the teachings of the gospel, are made to suffer everlasting torments, because they didn't know, what they couldn't know, and didn't do what they were never taught to do, and never thought of doing.

At the same time their eastern brethren of like pursuasion, schooled and schooling others in the Andovarian system of fire insurance, have had under consideration with equal soberness, the permissibility of a wavering belief which might be said to amount to a qualified doubt on the part of the insured, of some of the biblical accounts of natural and supernatural phenomena, without imparing his individual "*risk*," for example: Jonah's skirmish with the whale, Joshua's confidential relations with the sun, Elisha's body guard of bears, and the like.

It should be stated that this subject was not introduced in the interest of public or private morals, nor with the object of correcting any abuse of church privileges, for it was conceded by a fair minority, that it was possible for one to live uprightly and do a fair measure of good, even though so wide of the correct thing as to entertain private views of his own, upon these interesting questions.

But the real cause of the rumpus was both tempora and spiritual. It seems that the good old gentleman, who wound up a well spent life by creating the Andover benefic, was a strict literalist in respect to all biblical statements, and made their exactitude in holding and teaching, the condition of its maintenance, and to provide against any weakening upon doctrinal points, the professors in charge were required to be pulled back by the coat tails once in every five years to make sure that they had in nowise outgrown the good old garment which passed muster upon their initiation.

In a word, if they now hold to an *incorruptible* belief that Jonah swallowed the whale—that Joshua was too much for the sun—that Elisha invented bear's oil for bald heads, they were accounted fit to prune and bend the aspiring twigs of future theologic banyons, without endangering the escheatment of New England's hot bed of orthodoxy.

Another denominational body, which, for fear of mistakes in asking too much or too little by way of heavenly favors, is accustomed to have its prayers furnished, to hand, in clean letter-press, has lately met in convention and revised its regulation forms according to the latest advices from "over the river," whereby some rather tough words are omitted and substitutions made more in harmony with modern ideas and the usages of polite society, whil'st enough others to save old seed for new crops, keep right on with their old fashioned harangues and supplications, asking measureless quantities of special grace, and telling God how big he is and what poor miserable worms of the dust *they* are, and how they have a bare chance of being saved through his ill judged mercy, when in common justice they ought to be eternally dammed.

*And this is called worship !*

